# Weight and body mass index in relation to irradiated volume and to overall survival in patients with oropharyngeal cancer: a retrospective cohort study

**DOI:** 10.1186/1748-717X-9-160

**Published:** 2014-07-22

**Authors:** Sandra Ottosson, Karin Söderström, Elisabeth Kjellén, Per Nilsson, Björn Zackrisson, Göran Laurell

**Affiliations:** 1 Department of Clinical Sciences, Otorhinolaryngology, Umeå University, Umeå, Sweden; 2 Department of Radiation Sciences, Oncology, Umeå University, Umeå, Sweden; 3 Department of Oncology and Radiation Physics, Skåne University Hospital, Lund University, Lund, Sweden; 4 Department of Surgical Sciences, Otolaryngology and Head & Neck Surgery, Uppsala University, Uppsala, Sweden

**Keywords:** Oropharyngeal cancer, Treated volume, Weight loss, Body mass index, Survival

## Abstract

**Background:**

Weight loss is a common problem in patients with Squamous Cell Carcinoma of the Head and Neck (SCCHN) treated with radiotherapy (RT). The aims of the present study were to determine if treated volume (TV), as a measure of the radiation dose burden, can predict weight loss in patients with oropharyngeal cancer and to analyze weight loss and body mass index (BMI) in the same patient group in relation to 5-year overall survival.

**Methods:**

The ARTSCAN trial is a prospective, randomized, multicenter trial in patients with SCCHN. Nutritional data from the ARTSCAN trial were analyzed retrospectively using univariate and multivariate statistical methods based on information on percentage weight loss from the start of RT up to five months after the termination of RT (study cohort 1, n = 232) and information on patients’ BMI at the start of RT (study cohort 2, n = 203). TV was defined as the volume of the patient receiving at least 95% of the prescribed dose. TV_64.6 Gy_ encompasses macroscopic tumor and TV_43.7 Gy_ elective lymph nodes of the neck.

**Results:**

TV_64.6 Gy_ and TV_43.7 Gy_ were both significantly correlated with higher weight loss up to five months after the termination of RT in study cohort 1 (*p* < 0.001 for both). BMI at the start of RT was shown to be a prognostic factor for 5-year overall survival in study cohort 2 but weight loss was not. The hazard ratios and 95% confidence intervals were 3.78 (1.46–9.75) and 2.57 (1.43–4.62) in patients with underweight and normal weight, respectively.

**Conclusions:**

TV can predict weight loss during RT in patients with oropharyngeal cancer regardless of clinical stage. A high BMI (>25 kg/m^2^) at the start of RT is positively associated with survival in patients with oropharyngeal cancer.

## Background

Weight loss is a common problem in patients with Squamous Cell Carcinoma of the Head and Neck (SCCHN) and this weight loss has a number of different etiologies [1]. The tumor itself can affect nutritional status both by its location and through metabolic alterations [2-5]. In addition, the toxic effects of oncologic treatment such as radiotherapy (RT) alone or in combination with surgery and/or anticancer drugs [6,7] can affect food intake [8-11] and thus increase the risk of weight loss [8,9,11-13].

Weight loss is one of the main characteristics of malnutrition [14]. However, not all patients with weight loss will develop malnutrition. In clinical practice, weight loss can be used together with body mass index (BMI) and information about eating difficulties to find patients at risk of nutritional deterioration [15]. It is recommended that nutritional screening should be initiated before the start of RT to find patients at risk and in need of nutritional interventions. Because the treatment for SCCHN can lead to further nutritional impairment, predictive factors for anticipated weight loss and nutritional decline during RT might be important pieces of information that can be obtained during patient history. Research results available today on patients with SCCHN suggest that tumor site, clinical stage, and use of chemo-radiotherapy are factors that can predict a significant weight loss during RT [16-18]. When assessing the different tumor sites, patients with oropharyngeal cancer have been shown to lose more weight compared to patients with tumors at other sub-sites [19-21]. In recent years, a number of publications have addressed the dose-volume relationship in different organs at risk (OARs) that affect swallowing function and subsequently might lead to weight loss [22-25]. This has also been shown for different tumor sub-sites, e.g. oropharyngeal cancer [26-29].

The ARTSCAN trial is a Swedish multicenter, randomized, controlled clinical trial in patients with SCCHN comparing conventional fractionation (CF) with accelerated fractionation (AF) [30]. In previously published results from the ARTSCAN trial, we found that patients with oropharyngeal cancer lost significantly more weight during and after RT compared to patients with tumors of the larynx and oral cavity [19]. In the present study, we investigated the relationship between the treated volume (TV) and weight loss in a homogenous cohort at risk of weight loss who were treated with RT. TV was used as a measure of the radiation dose burden as defined by the International Commission on Radiation Units & Measurements (ICRU, reports 50 and 62) [31,32]. This study was performed retrospectively using nutritional data from the subgroup of patients with oropharyngeal cancer in the ARTSCAN trial.

Earlier studies have investigated the relationship between different nutrition-related factors and survival in patients with SCCHN [18,33-42]. The results tend to vary, but the trend in the data suggests that BMI might have a more prominent role in survival than weight loss per se. Both McRackan et al. [40] and Pai et al. [33] showed, for example, that patients with SCCHN who had an initial BMI over 25 kg/m^2^ had a higher chance of survival. The research available for weight loss suggests that weight loss prior to treatment [35-37] or weight loss in patients with recurrent disease [38,39] might have a negative influence on survival. Given the current research, the relation between BMI and survival needs to be further established in different sub-groups of SCCHN. In addition, the correlation between survival and weight loss during RT needs more thorough investigation. In the present study, this is explored in the same cohort of patients with oropharyngeal cancer as described above.

### Objectives

The aims of the present study were to:

• Analyze if TV can predict weight loss in patients with oropharyngeal cancer and thereby provide information on patients at risk of malnutrition and in need of special nutritional surveillance.

• Analyze weight loss and BMI in patients with oropharyngeal cancer in relation to 5-year overall survival.

## Methods

### Patients

This nutritional study is based on data from the ARTSCAN trial conducted between the years 1998 and 2006. Seven hundred and fifty patients (age >18 years) with non-distant metastatic SCCHN, i.e. cancer in the oral cavity, oropharynx, hypopharynx, or larynx, were included in the ARTSCAN trial at 12 treatment centers across Sweden. Patients specifically with oropharyngeal cancer were selected for the present study (n = 357). All patients gave written consent before randomization, and the study was approved by the local ethics committees (Dnr 07-023 M/FEK98-139). For more information about the ARTSCAN trial, see Zackrisson et al. [30].

### Data

Medical, treatment, and follow-up data were collected prospectively in the ARTSCAN trial up to 5 years after the termination of RT in surviving patients. After 5 years, survival was followed through the Swedish population registry. Details about the patient material, methods, and structure for data collection in the ARTSCAN trial are described elsewhere [30,43].

#### Radiotherapy

All patients received CT-based three-dimensional conformal radiotherapy (3-DCRT) and/or intensity-modulated radiotherapy (IMRT) with dose prescriptions according to the recommendations of the ICRU [31,32]. A more detailed presentation of the RT and the quality assurance process of the trial have been reported earlier [30,43]. In short, 68 Gy was prescribed to the known tumor in the oropharynx and metastatic cervical lymph node/nodes. An additional adjuvant dose of 46 Gy was prescribed to elective lymph nodes of the neck.

##### Absorbed doses and volumes

TV, as a measure of the radiation dose burden, was defined as the volume (cm^3^) of the patient receiving at least 95% of the prescribed dose [31,32]. TV_64.6 Gy_ encompasses macroscopic tumor and TV_43.7 Gy_ elective lymph nodes of the neck.

#### Nutritional data

Weight was measured every week during RT, at 4-6 weeks after RT, every 3 months for the first 2 years after RT, and thereafter every 6 months up to 5 years. In the present study, percent weight change up to 5 months after the termination of RT was calculated using weight at the start of RT as the reference point. This time-period for weight loss was chosen on the basis of previously published results from the ARTSCAN trial showing a nadir of weight loss for the entire cohort at 5 months after the termination of RT [19]. Weight loss was used either as a continuous variable or was dichotomized (<10% and ≥10%). Patients with ≥10% weight loss were defined as at risk of nutritional deterioration [44].

Height was gathered through the medical records and used to calculate BMI (kg/m^2^). The following cut-off values were used in the analyses: underweight BMI < 20 kg/m^2^ (or BMI < 22 kg/m^2^ if ≥70 years of age), normal weight BMI 20–25 kg/m^2^ (or BMI 22–27 kg/m^2^ if ≥70 years of age), and overweight/obesity BMI >25 kg/m^2^ (or BMI >27 kg/m^2^ if ≥70 years of age) [15,45].

The patients could be classified into the following three groups according to type of nutritional support administered during the study: oral intake (with or without nutritional counseling and/or oral nutritional supplements), tube feeding (TF) using nasogastric feeding tube or percutaneous endogastric gastrostomy, and parenteral nutrition. The nutritional support was administered when needed according to local guidelines at each participating center. Use of TF and parenteral nutrition was registered in the study protocol, and information on the use of TF at the start and end of RT was used in the present study.

### Statistical analysis

The Statistical Package for the Social Sciences (SPSS) version 21.0 and R version 2.15.2 software packages were used for the statistical analyses. All tests were two-sided and a *p*-value less than 0.05 was considered significant.

The independent samples *t*-test, one-way between-groups ANOVA, and Fisher’s exact test were used for univariate analyses. A multiple linear regression analysis was used as the multivariate alternative, and variables that were statistically significant in the univariate analyses were included as the independent variables in the model. The dependent variable (weight change) was numerical and the independent variables were either numerical or dichotomized. The unstandardized regression coefficients (B) represent an increase (positive values) or a decrease (negative values) in weight (percentage points). A regression model based on cubic splines was used to illustrate the nonlinear correlation between weight loss and TV [46].

For the survival analyses, time to death was calculated from the start of RT up to 5 years in surviving patients. The Kaplan–Meier estimators for the BMI and weight groups were compared using the log rank test. The Cox proportional hazard model was used to calculate the hazard ratios (HRs) and their 95% confidence intervals (CI). The variable of interest (BMI) was included in the adjusted model together with potential confounder variables related to patient, tumour, and treatment characteristics. Variables that did not meet the assumption of proportional hazard were used as strata in the model.

## Results

### Eligibility and patient characteristics

Three hundred and fifty-seven patients were diagnosed with oropharyngeal cancer in the ARTSCAN trial. A per protocol analysis was performed, and percent weight change between the start of RT up to 5 months after the termination of RT was available for 232 patients (65.0%), and these patients were referred to as study cohort 1. The patients not included in this cohort were due to death (n = 23), loss of follow-up or residual or recurrent disease (n = 30), or missing data (n = 72). BMI at the start of RT was available for 203 patients (56.9%) and this group was named study cohort 2. Patient, tumor, and treatment characteristics for all patients with oropharyngeal cancer and for patients in study cohorts 1 and 2 are presented in Table 1.

**Table 1:** Characteristics of patients with oropharyngeal cancer (n = 357) from the ARTSCAN trial as well as for study cohorts 1 (n = 232) and 2 (n = 203)

	**Patients with oropharyngeal cancer, n (%)**	**Study cohort 1, n (%)**	**Study cohort 2, n (%)**
*Age*			
* Median (min, max)*	58 (32, 86)	57 (32, 86)	57 (32, 86)
*<65 years*	275 (77.0)	184 (79.3)	160 (78.8)
*≥65 years*	82 (23.0)	48 (20.7)	43 (21.2)
*Sex*			
*Male*	267 (74.8)	173 (74.6)	154 (75.9)
*Female*	90 (25.2)	59 (25.4)	49 (24.1)
*Clinical stage*			
*I*	10 (2.8)	8 (3.4)	9 (4.4)
*II*	20 (5.6)	16 (6.9)	13 (6.4)
*III*	84 (23.5)	54 (23.3)	41 (20.2)
*IV*	243 (68.1)	154 (66.4)	140 (69.0)
*Surgery*			
*Yes*	166 (46.5)	103 (44.4)	106 (52.2)
*No*	191 (53.5)	129 (55.6)	97 (47.8)
*Conventional fractionation*	178 (49.9)	117 (50.4)	106 (52.2)
*Accelerated fractionation*	179 (50.1)	115 (49.6)	97 (47.8)
*Number of patients*	n = 357	n = 232	n = 203

### Predictive factors for weight loss

Weight change from the start of RT up to 5 months after the termination of RT in study cohort 1 (n = 232) was analyzed together with tumor- and treatment-related factors in univariate analyses (Table 2). The three factors significantly related to weight loss were the use of TF at the start of RT (*p* = 0.024), TV_64.6 Gy_ (*p* < 0.001), and TV_43.7 Gy_ (*p* < 0.001).

**Table 2:** Predictive factors for weight loss at 5 months after the termination of radiotherapy (RT) is shown in relation to weight at the start of RT (n = 232)

			**n (%)**	**Weight loss % ** **mean ± SD**	*p*-value*	**Missing data**
***Tumor-related factors***	*Clinical stage*	*I + II*	24 (10.3)	-12.96 ± 7.36	0.643	-
		*III + IV*	208 (89.7)	-13.74 ± 7.95		
***Treatment-related factors***	*Treatment type*	*CF*†	117(50.4)	-12.96 ± 7.86	0.170	-
		*AF*‡	115 (49.6)	-14.38 ± 7.86		
	*Surgery*	*Yes*	101 (43.5)	-13.97 ± 7.82	0.601	-
		*No*	131 (56.5)	-13.42 ± 7.94		
	*Tube feeding at the start of RT*	*Yes*	12 (5.2)	-8.70 ± 8.16	**0.024**	2
		*No*	218 (94.8)	-13.97 ± 7.79		
	*Tube feeding at the end of RT*	*Yes*	103 (46.0)	-12.82 ± 8.56	0.126	8
		*No*	121 (54.0)	-14.45 ± 7.36		
			**Weight loss%**			
			**<10%**	**≥10%**		
	*Treated volume (cm*^*3*^*), mean ± SD*	*TV*_*64.6Gy*_	494.00 ± 190.43	621.00 ± 285.54	**<0.001**	2
	*Treated volume (cm*^*3*^*), mean ± SD*	*TV*_*43.7Gy*_	1247.66 ± 481.94	1583.26 ± 610.24	**<0.001**	2

*A *p*-value less than 0.05 was considered significant as determined by the independent samples *t*-test. †Conventional fractionation. ‡Accelerated fractionation.

Information about TF use at the start of RT was available for 230 patients (missing n = 2). Twelve patients (5.2%) received TF at the start of RT. Information about TV was available for 230 patients (missing n = 2). The average volumes for TV_64.6 Gy_ and TV_43.7 Gy_ were 580 cm^3^ ± 265 cm^3^ and 1475 cm^3^ ± 592 cm^3^, respectively.

A multiple linear regression model was used to analyze how much of the variation in weight loss that could be explained by the variables statistically significant in the univariate analyses as well as the relation between the dependent variable (weight loss up to 5 months after the termination of RT) and each of the independent variables. Because of the close relation between TV_64.6 Gy_ and TV_43.7 Gy_, two different analyses were performed. In the first analysis, TF at the start of RT (yes/no) and TV_64.6 Gy_ (numerical) were included in the model (n = 228). Clinical stage (I + II or III + IV) was also included as a confounding factor for TV. The coefficient of determination (*R*^2^) was 0.084 (*p* < 0.001). The use of TF at the start of RT and TV_64.6 Gy_ were shown to be significantly predictive for weight loss up to 5 months. Patients without TF at the start of RT lost more weight than patients with TF (B = 4.946, *p* = 0.03), and patients treated with larger TV_64.6 Gy_ had a significantly greater weight loss up to 5 months after RT (B = -0.008, *p* < 0.001). The same multiple linear regression analysis was performed with TV_43.7 Gy_ (n = 228). *R*^2^ was 0.142 (*p* < 0.001), and the presence of TF at the start of RT and TV_43.7 Gy_ were shown to be significantly predictive for weight loss up to 5 months. Patients without TF at the start of RT lost more weight than patients with TF (B = 5.250, *p* = 0.017), and patients treated with larger TV_43.7 Gy_ had a significantly larger weight loss up to 5 months after RT (B = -0.005, *p* < 0.001). Clinical stage was not significantly predictive for weight loss in either of the multivariate models (*p* = 0.449 and p = 0.138, respectively).

**Figure 1:**
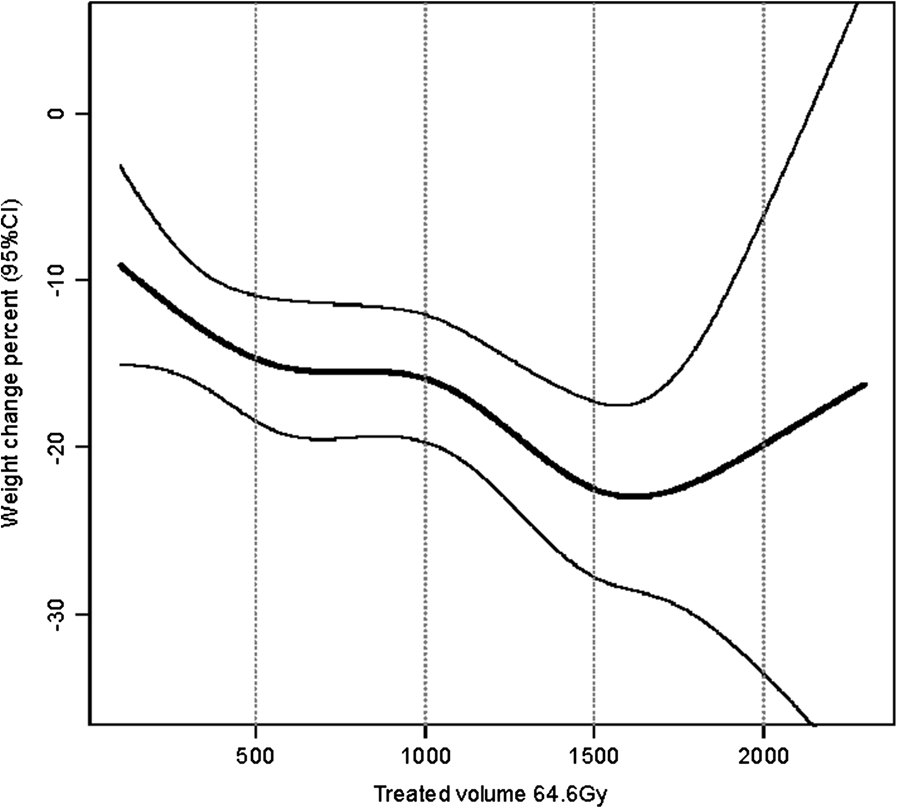
**Nonlinear correlation between treated volume (TV**_**64.6 Gy**_**) and weight change.** Regression model based on cubic splines for weight change in percent (95% CI) from the start of RT up to 5 months after RT with TV_64.6 Gy_ and controlling for clinical stage and tube feeding at the start of RT (n = 228).

**Figure 2:**
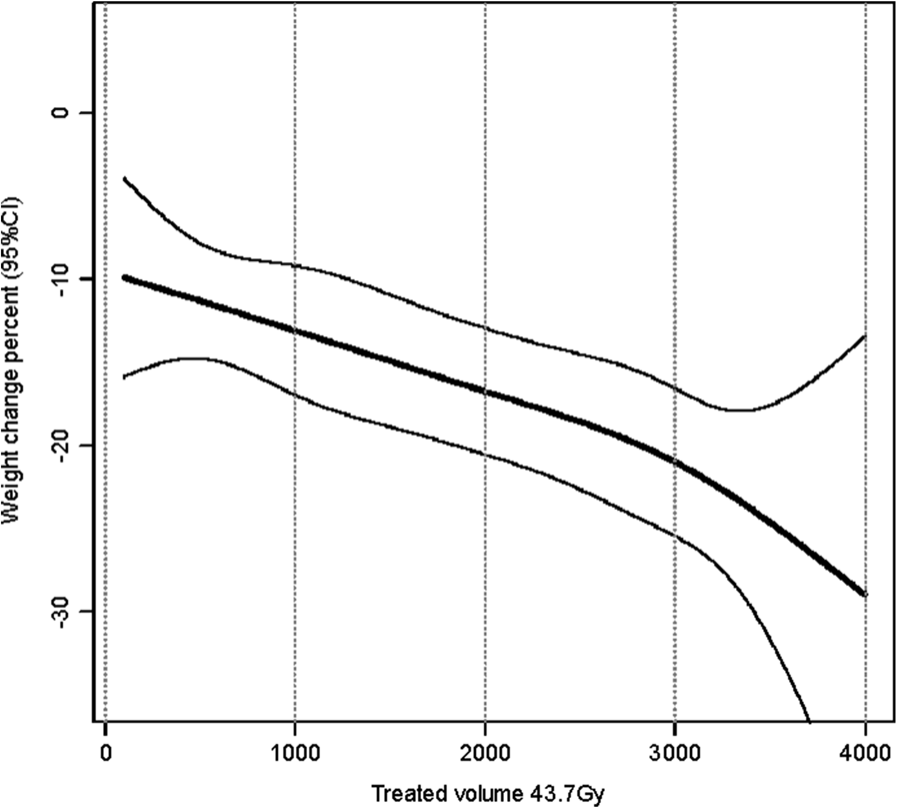
**Nonlinear correlation between treated volume (TV**_**43.7 Gy**_**) and weight change.** Regression model based on cubic splines for weight change in percent (95% CI) from the start of RT up to 5 months after RT with TV_43.7 Gy_ and controlling for clinical stage and tube feeding at the start of RT (n = 228).

A regression model based on cubic splines was used to illustrate the nonlinear relationship between weight change up to 5 months after RT and TV_64.6 Gy_ (Figure 1) and TV_43.7 Gy_ (Figure 2) while also controlling for clinical stage and TF at the start of RT (n = 228). For TV_43.7 Gy_ (Figure 2), the relation between weight and TV displayed an almost linear shape. However, another type of relation was found between weight and TV_64.6 Gy_ (Figure 1). Between 500 cm^3^ and 1000 cm^3^, the weight seemed to stabilize, and for volumes above 1000 cm^3^ the volume effect on weight loss increased more steeply. Above 1500 cm^3^, the 95% CI became wider due to fewer events.

### Weight and BMI in relation to 5-year overall survival

Percent weight change from the start of RT up to 5 months after the termination of RT was dichotomized into <10% (n = 74) and ≥10% (n = 158) and analyzed in relation to 5-year overall survival in study cohort 1 (n = 232). The 5-year overall survival rates for the two weight-change groups were 77.0% and 74.1%, respectively (log rank: *p* = 0.708).

According to the three BMI classifications, 8.4% of the patients were underweight, 33.0% were normal weight, and 58.6% were overweight or obese at the start of RT. Patients with overweight or obesity had a significantly higher percent weight loss up to 5 months (15.3%) compared to patients with normal weight (11.8%, p = 0.022) or underweight (4.9%, p < 0.001).

**Figure 3:**
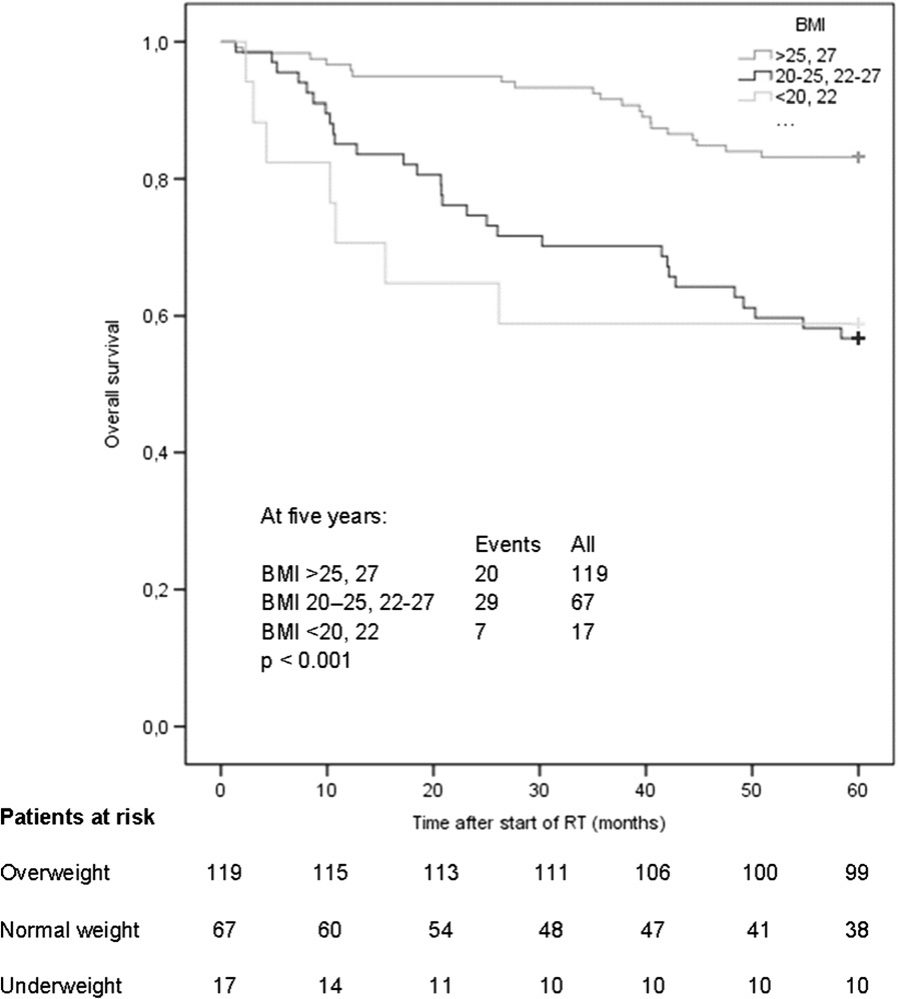
**Overall survival by body mass index (BMI) category.** Kaplan–Meier plot of overall survival by BMI category at the start of RT (n = 203). The following cut-off values were used in the analyses: underweight BMI < 20 kg/m^2^ (or BMI < 22 kg/m^2^ if ≥70 years of age), normal weight BMI 20–25 kg/m^2^ (or BMI 22–27 kg/m^2^ if ≥70 years of age), and overweight/obesity BMI >25 kg/m^2^ (or BMI >27 kg/m^2^ if ≥70 years of age).

BMI at the start of RT was analyzed in relation to 5-year overall survival in study cohort 2 (n = 203). There was a significant difference in 5-year overall survival between patients with different BMI at the start of RT (log rank: *p* < 0.001) (Figure 3). Patients who were underweight and normal weight at the start of RT had lower survival rates (58.8% and 56.7%, respectively) than patients who were overweight or obese (83.2%). For the unadjusted Cox regression analysis, the HRs and 95% CI were 3.31 (1.40–7.83) (*p* = 0.006) and 3.07 (1.74–5.44) (*p* < 0.001) for patients with underweight or normal weight, respectively. In the adjusted Cox regression, the following variables were included together with BMI: age (numerical value), sex (male/female), clinical stage (I + II or III + IV, used as strata), RT schedule (CF/AF), and surgery (yes/no). The HRs and 95% CI were 3.78 (1.46–9.75) (*p* = 0.006) and 2.57 (1.43–4.62) (*p* = 0.002) in patients with underweight and normal weight, respectively.

## Discussion

It is well known that RT for oropharyngeal cancer can lead to nutritional deterioration. The present study has retrospectively explored TV as a predictive factor for weight loss as well as weight loss and BMI in relation to 5-year overall survival in a cohort consisting of patients with oropharyngeal cancer under nutritional surveillance. A major finding was that TV, as a measure of the radiation dose burden, was of greater predictive value than clinical stage for weight loss from the start of RT up to 5 months after the termination of RT. Another finding was that patients with overweight or obesity had better 5-year overall survival when compared to patients with underweight and normal weight. The same relation could not be shown for weight loss and 5-year overall survival.

A higher degree of treatment toxicity due to a larger radiation dose burden might be a possible explanation as to why patients in the present study who were treated with larger TV had significantly greater weight loss. It is obvious that the larger the TV the higher the risk for exceeding the dose volume constraints in a number of OARs. Despite increasing knowledge on such parameters, there is still a lack of detailed information for several OARs concerning the interrelationship and risks from combined dose-volume responses. For that reason, the TV in a defined set of organs might be useful as a surrogate parameter. This might be of particular use for weight loss, which is highly multi-factorial. Dysphagia is known to be related to the prescribed dose in specific OARs [22-25] and in addition, other side effects of RT such as mucositis and xerostomia [1,6] are known to be related to the irradiated volume. The size of the TV alone might thus predict weight loss, regardless of included OARs, through both locally (increased toxicity response) and systemic effects [47]. It can be speculated that inflammatory mediators produced in response to RT could have a negative influence on the physiological regulation of the appetite thus leading to decreased food intake [2]. Hence, the data from this site-specific cohort support the notion that TV can be used during RT planning to predict which patients are at risk of weight loss and, therefore, are in need of special nutritional surveillance during RT.

The finding that larger TV can predict patients at risk for increased weight loss during and after RT prompted us to further investigate whether there was a specific cut-off for the analyzed TVs that could be used in a clinical setting. For TV_64.6 Gy_, the volume effect on weight loss increased for volumes above 1000 cm^3^. However, as the 95% CI became wider above 1500 cm^3^ and therefore more difficult to interpret, more studies are needed in order to identify if there exists any specific volume cut-off with clinical relevance.

The results from the current study cannot confirm that weight loss per se is a negative prognostic factor in patients with oropharyngeal cancer. In fact, patients with a high BMI showed the largest weight loss but still had a better 5-year overall survival. This indicates that pretreatment BMI can be used as a prognostic indicator for 5-year overall survival in patients with oropharyngeal cancer but weight loss in connection to treatment cannot. However, it is difficult to come to any conclusion regarding objective nutritional measures as prognostic indicators for survival in SCCHN because of the diverse nature of the available literature in terms of both results and study design [18,33-42]. Still, the results from the present study for BMI confirm the results of previous research [33,40,41]. The relation between a higher BMI and greater survival might be explained by the larger amount of adipose tissue available to overweight and obese patients. It seems as though the amount of fat mass might determine the amount of lean body mass that can be spared during negative energy balance [48], and this might be of significant clinical value.

Interpretation of the results in this study showing a significant relationship between BMI and survival in patients with oropharyngeal cancer must take confounding factors into consideration. Previous well-known prognostic factors for survival in oropharyngeal cancer are stage, human papilloma virus (HPV) infection, and smoking [49,50]. Prevalence of HPV infection and smoking habits could not be controlled for in the present study because this information was not available for the study cohort. However, when controlling for smoking in a multivariate analysis, McRackan et al. [40] still found a significant impact of BMI on survival. Because the majority of patients with oropharyngeal cancer are currently HPV-positive, it is less probable that HPV is a confounder for BMI. However, the relation between HPV and BMI needs to be better assessed.

One limitation of this study is the number of patients available for analysis. The information on BMI and weight was reduced primarily due to missing data, which implies a potential selection bias of patients available for analysis. In addition, few patients received TF at the start of RT and this should be considered when interpreting the result from the regression analysis. However, one of the strengths of the current study was that all participants had oropharyngeal cancer making the cohort homogenous.

The present study has important clinical implications. TV, defined as the volume of the body encompassed by the 95% isodose of the prescribed dose [31,32], was registered in the ARTSCAN database and was thus chosen in this study as a measure of the radiation dose burden to the patient. The TV might not always be available for the pretreatment decision of a nutritional intervention. With highly conformal RT, the result for the TV might well be transferred to the delineated target, i.e. the planning target volume (PTV). For example, Mallick and coworkers [20] studied PTV instead of TV in relation to weight loss during RT and found a significant impact of larger PTV on weight loss. In this study, the conformity index [32] of TV_64.6 Gy_/PTV_68 Gy_ was 1.89 ± 0.59 and the conformity index of TV_43.7 Gy_/PTV_46 Gy_, was 2.03 ± 0.45. This suggests that both the PTV and TV can be used as predictors for weight loss and highlights the importance of concise target delineation. Also, based on the results of the present study it seems desirable to strive for a high BMI before initiating RT. In addition, future prospective studies should investigate whether nutritional interventions can improve outcomes in patients with less beneficial nutritional status at the start of RT.

## Conclusions

The results of the present study showed that larger TV_64.6 Gy_ and TV_43.7 Gy_ were associated with increased weight loss up to 5 months after the termination of RT regardless of clinical stage. Therefore, TV can be used to identify patients at risk of malnutrition and in need of special nutritional surveillance during RT. The results also showed that BMI at the start of RT can be used as a prognostic factor for 5-year overall survival.

## Competing interests

The authors declare that they have no competing interests.

## Authors’ contributions

All authors were involved in the study design (BZ, EK and PN for the initial ARTSCAN trial design); SO and KS were responsible for writing the manuscript; and PN, EK, BZ, and GL were responsible for critical revision of the manuscript. SO performed the statistical analyses. All authors read and approved the final version of the manuscript.
